# Occurrence and Identification of *Ixodes ricinus* Borne Pathogens in Northeastern Italy

**DOI:** 10.3390/pathogens10091181

**Published:** 2021-09-13

**Authors:** Michela Bertola, Fabrizio Montarsi, Federica Obber, Graziana Da Rold, Sara Carlin, Federica Toniolo, Elena Porcellato, Christian Falcaro, Valeria Mondardini, Silvia Ormelli, Silvia Ravagnan

**Affiliations:** 1 Istituto Zooprofilattico Sperimentale delle Venezie, Viale dell’Università 10, Legnaro, 35020 Padua, Italy; fmontarsi@izsvenezie.it (F.M.); FObber@izsvenezie.it (F.O.); GDaRold@izsvenezie.it (G.D.R.); SCarlin@izsvenezie.it (S.C.); FToniolo@izsvenezie.it (F.T.); EPorcellato@izsvenezie.it (E.P.); cfalcaro@izsvenezie.it (C.F.); sormelli@izsvenezie.it (S.O.); sravagnan@izsvenezie.it (S.R.); 2 UOC Malattie Infettive Ospedale San Martino, 32100 Belluno, Italy; valeriamondardini@gmail.com

**Keywords:** tick-borne pathogens, surveillance, co-infection, prevalence, *Rickettsia* spp., *Borrelia* spp, *Anaplaspa phagocitophilum*, *Neoerlichia mikurensis*, *Babesia venatorum*

## Abstract

In Europe, *Ixodes ricinus* is the main vector for tick-borne pathogens (TBPs), the most common tick species in Italy, particularly represented in pre-alpine and hilly northern areas. From 2011 to 2017, ticks were collected by dragging in Belluno province (northeast Italy) and analyzed by molecular techniques for TBP detection. Several species of *Rickettsia* spp. and *Borrelia* spp. *Anaplaspa phagocitophilum*, *Neoerlichia mikurensis* and *Babesia venatorum*, were found to be circulating in the study area carried by *I. ricinus* (n = 2668, all stages). Overall, 39.1% of screened pools were positive for at least one TBP, with a prevalence of 12.25% and 29.2% in immature stages and adults, respectively. Pathogens were detected in 85% of the monitored municipalities, moreover the presence of TBPs varied from one to seven different pathogens in the same year. The annual TBPs prevalence fluctuations observed in each municipality highlights the necessity of performing continuous tick surveillance. In conclusion, the observation of TBPs in ticks remains an efficient strategy for monitoring the circulation of tick-borne diseases (TBDs) in a specific area.

## 1. Introduction

Pathogens transmitted by ticks (TBPs) are responsible for the majority of the vector-borne diseases in temperate North America, Europe, and Asia [1]. The economic impact of tick-borne diseases (TBDs) is significant and increases every year but unfortunately the combined public health impact of TBDs remains mostly unquantified [1,2].

Given the fact that TBDs could severely concern both human and animal health, surveillance programs for TBDs and TBPs, based on harmonized One Health approaches, have been implemented in several European countries in recent years [3,4]. 

In Europe, *Ixodes ricinus* (Acari: Ixodidae) is considered one of the primary vectors of multiple pathogens that affect human and animal health [5]. These pathogens (i.e., virus, bacteria, and protozoa) circulate in enzootic cycles, alternating between ticks and suitable animal hosts. In addition, infected *I. ricinus* can transmit TBPs to humans, causing several TBDs [6,7]. 

The most frequently diagnosed zoonoses transmitted by *I. ricinus* are Lyme borreliosis (LB) and tick-borne encephalitis (TBE) [8,9,10]. Lyme borreliosis (LB) is caused by three species of spirochetes consisting of the *B. burgdorferi* sensu lato (s.l) complex (*Borrelia burgdorferi* sensu stricto (s.s), *B. afzelii* and *B. garinii*). Tick-borne encephalitis virus (TBEv, Flaviviridae) is described as the agent of the human TBE disease. 

A wide range of TBPs responsible for human disease have been detected in European *I. ricinus*, including *Rickettsia* (*Rickettsia helvetica*, *R. monacensis*, *R. raoultii*, *R. limoniae*), *Ehrlichia* spp., *Borrelia* (*B. valaisiana*, *B. lusitaniae* and *B. miyamotoi*), *Babesia* (*Ba. microti*, *Ba. divergens*, *Ba. duncani*, and *Ba. venatorum*), *Anaplasma phagocytophilum*, and *Neoehrlichia mikurensis* [6,11,12,13,14,15,16,17,18]. 

Lyme borreliosis is associated with numerous TBPs species, although the pathogenicity of *B. lusitaniae* and *B. valaisiana* is still unclear [19,20,21].

*Anaplasma phagocytophilum* is considered an emerging pathogen which causes an underdiagnosed clinical manifestation, namely human granulocytic anaplasmosis (HGA) [22], although many of its strains are non-pathogenic to humans [22,23].

*Rickettsia monacensis* has been shown to cause a Mediterranean spotted fever-like illness in humans in different European countries, including Italy [24]. Likewise, *R. helvetica* was associated with human illness, and three cases of a mild form of human rickettsiosis were attributed to *R. helvetica* in northern Italy through serological analyses [25,26].

*Neoehrlichia mikurensis* was discovered very recently and even though it is considered a newly emerging TBP, it may be underestimated so far. The first case of human infection was reported in 2010 in Switzerland [27] and eighteen cases of human infection have been reported in Europe to date [28].

Other TBPs associated with human disease are *Ba. venatorum* [29,30,31,32,33,34,35,36,37] and *B. miyamotoi,* which were identified in *I. ricinus* both in several central European countries and Italy [38]. Recently, *B. miyamotoi* has been recognized as the agent of a non-specific febrile syndrome often misdiagnosed as acute Lyme disease [39].

The distribution of TBPs is primarily related to tick density and to the availability of animal reservoirs. During the last decades, the distribution of *I. ricinus* in Europe has expanded as a result of multipartite interactions between climatic, ecological, landscape and anthropogenic drivers [40]; these factors, impacting on the transformations of biotopes, affect the available habitats for ticks and encourage the host–tick–pathogen interaction which leads to a higher risk of infection with TBPs [5].

In the last few years, recreational outdoor activities have greatly increased their consistency, and an increase in the number of tick bites has been reported in urban and suburban areas, such as city parks or suburban forests [6,7,41].

The emerging crucial meeting point between wild animals, humans, pets, and ticks is given by the peri-urban recreational areas [6], in which the evaluation of TBP distribution needs a constant update to maintain the awareness of TBDs.

The territory of northeastern Italy, due to its climatic characteristics and host availability (high diversity and availability of both wild and domestic animals), provides favorable ecological conditions for *I. ricinus* proliferation, the most abundant tick in this area [42]. 

For years, a high tick abundance, TBP circulation, and TBD cases have been reported in the Veneto region; in particular, the Belluno province is historically considered an endemic area for TBPs [43,44,45], and it will be classified as a risk area soon [46,47,48]. 

In fact, though Italy historically reported a low incidence rate of LB, ranging from 0.001 to 0.02 new cases per 100,000 inhabitants [49,50], from 2006 to 2019 the Veneto region reported 1259 LB cases, with an increasing trend of the annual mean incidence over five years (2015–2019) of 1.916/100,000 inhabitants [48]. Remarkably, in Belluno province during the period of 2015–2017, 76 cases were observed (unpublished data provided by Local Public Health Dep. Dolomiti, Veneto Region) and the trend seems to be constantly growing; in fact, in 2019 40 LB cases were reported at the Belluno Hospital (recognized as Regional Reference Center for TBDs (unpublished data).

Likewise, the annual TBE incidence rate in this province (4.9 per 100,000 inhabitants) is the highest recorded in the municipalities [46]. For this reason, people practicing both recreational and professional outdoor activities, such as forestry workers, farmers, veterinarians, military workers, and outdoor workers are potentially exposed to tick bite risk and possible TBP infections. 

This investigation began after several tick bite advisories from forestry workers and outdoor workers (professional hazard) and the cooperation with Carabinieri Corps and Forestry Team (Belluno department) carried out the monitoring activities and tick sampling during the years 2011–2017. 

The aim of this study was to estimate the occurrence of TBPs in *I. ricinus* ticks collected in Belluno province.

## 2. Results

### 2.1. Ticks Surveillance

The study area involved 20 municipalities in Belluno province, in which 39 monitored sites were found positive for tick presence, corresponding to 95.12% of total sites investigated during the seven years of study. 

During the sampling period, 187 dragging transects of 100 m^2^ each were performed (total 18,700 m^2^) within 39 sites positive for ticks monitored 1 to 38 times. In total, 2668 ticks were collected, all belonging to *I. ricinus*. Nymphs (2062) and larvae (331) were the most frequently collected development stages, followed by adults (147 males and 128 females, total 275) and immature ticks, with an adult ratio of 8.7:1 (Table 1 and Table A1).

Collected specimens were pooled according to their life stage, sex, date, and sampling site, resulting in a total of 596 pools (25, 296 and 275 pools for larvae, nymphs, and adults, respectively).

A considerable variability in tick numbers per collection (1 to 126 ticks/collection, median = 5) was seen throughout the study period, in addition to two peaks in activity of *I. ricinus* recorded in spring (May–June) and autumn (October–November). The peak of tick presence was characterized from May (n total = 801, 30.0%) to June (n total = 657, 24.6%): 60.9% of nymphs and 73.8% of adults were collected, while the majority of larvae (81.3%) were collected in autumn (September, October).

Mean observed densities of *I. ricinus* per 100 m^2^ were 11.1, 1.8, and 1.5 per nymphs, larvae, and adults, respectively. The maximum number of nymphs per sampling was 160 specimens, followed by larvae (110) and adults (42). 

### 2.2. Tick-Borne Pathogen Detection

**Figure 1:**
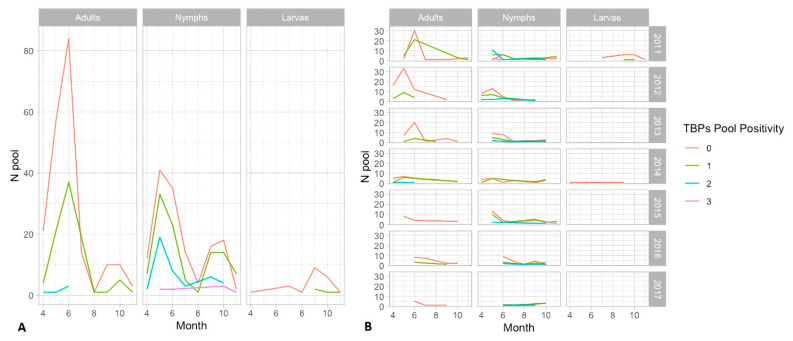
Number of pools negative (0) or positive for 1, 2, or 3 TBPs per tick stage collected monthly during 2011–2017 (**A**) and per year (**B**). Axes x (Month): 4 = April, 6 = June, 8 = August, 10 = October.

After molecular analysis of 2668 host-seeking ticks (all stages) belonging to 596 pools, 233 pools (39.1% of total tested) were positive for at least one TBP, ranging from one to three different pathogens. No tick was found to be infected with the TBE virus, although human TBE cases were reported in the same area during the surveillance period [46]. Tick density was higher in late spring (May–June), whereas the pool positivity rate (PPR) was higher in autumn (Table 2). Co-infected adults were collected from April to June, while multiple TBP presence in pooled nymphs was observed in late spring and autumn (Figure 1).

The highest TBP prevalence was detected in adult ticks (29.2) and was higher in females (32.0) compared to males (23.1). All tick stages were found to be infected with at least one TBP and *R. helvetica* and *N. mikurensis* were detected in each stage (Table 3).

The three most prevalent TBPs registered in adult ticks were *A. phagocytophilum* (7.6%), *R. helvetica* (6.9%), and *B. burgdoferi* s.s. (6.7%), followed by *N. mikurensis* (2.9%), *B. afzelii* (2.6%), *R. monacensis* (1.8%), and *B. valaisiana* (0.8%).

Despite adult ticks being more likely to be infected than nymphs with an overall prevalence of 29.2 (versus prevalence of 11.2 in nymphs) and higher prevalence for each TBP, in the latter a higher TBP diversity was detected, given that *B. garinii* and *Ba. venatorum* were detected only in nymphs. 

Molecular investigation revealed the presence of coinfection in adults (1.8%) and multiple TBP detection in pooled nymphs (16.9%).

Five adult ticks (1.8% of total) sampled in four different sites and periods were co-infected with two pathogen species (see Table 4 for details).

Eight nymph pools (composed of 10 individuals each) coming from four different sites located in three different municipalities during six samplings (2011, 2012, 2014, and 2015) were found to be positive for three TBPs, while 42 nymph pools (from 1 to 13 specimens) collected during 30 different samplings in eight municipalities during the whole studied period were positive with two TBPs. In one site (Col della Feda), two nymph pools collected the same day were positive at four different TBPs (i.e., *R. helvetica*, *B. garinii*, *B. burgdoferi* s.s., and *A. phagocytophilum*). All the TBPs, except *Ba. venatorum,* were detected in different combinations: *R. helvetica* and *A. phagocytophilum* were the most frequent combination (12 pools), followed by *R. helvetica* and *B. afzelii* (9 pools) and *R. helvetica* and *N. mikurensis* (5 pools) (Table 5).

**Figure 2:**
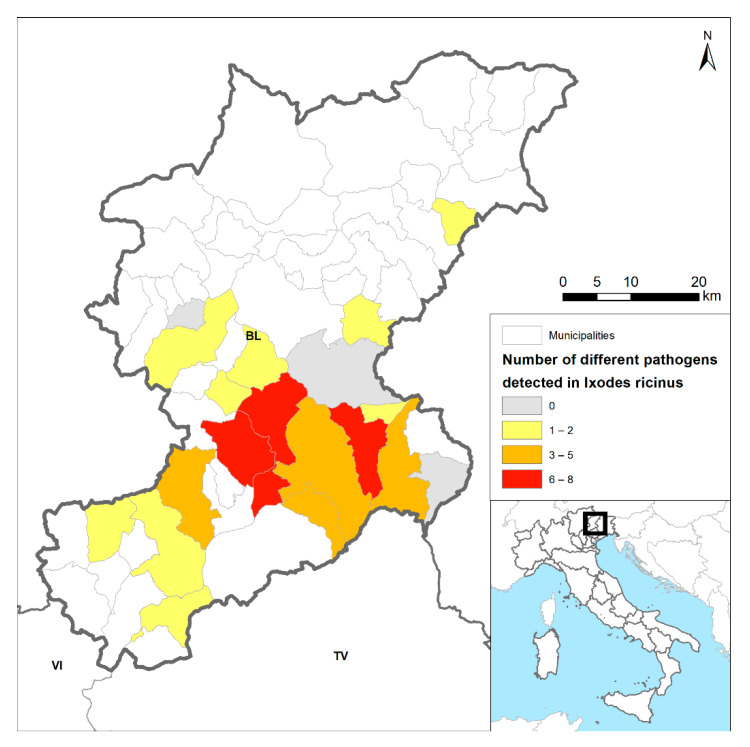
Occurrence of TBPs detected in monitored municipalities in Belluno province during 2011–2017.

All the 20 municipalities monitored tested positive for tick presence and among them, 17 (85%) tested positive for at least one TPBs during the study period (Figure 2). In three municipalities (Sedico, Sospirolo, Ponte nelle Alpi) the circulation of more than six TBPs was demonstrated. The highest diversity in TBPs was detected in the Sospirolo municipality, with eight different pathogens circulating in the whole study period and seven TBPs co-circulating in the same year.

Despite Sedico, Sospirolo, and Ponte nelle Alpi municipalities registering the biggest diversity in TBP species occurring per year, the highest TBP prevalence was reached in La Valle Agordina (100.0%), Ospitale di Cadore (50.0%), and Alpago (44.4%) (see Table 6). In municipalities monitored for several years, notable prevalence fluctuations can be observed (e.g., Limana registered a minimum TBP prevalence of 6.7 in 2016 and a maximum TBP prevalence of 100.0 in 2012) (see Table A1 for geographical details). In general, the mean prevalence of TBPs in Belluno municipalities during the study period was 21.7%.

During a retrospective study (unpublished data), *B. miyamotoi* presence was investigated in a subsample of stored ticks collected in Belluno province during 2011–2017. Of the 343 (57.5%) analyzed pools, 15 were positive for *B. miyamotoi* (details are available in the Appendix A, Table A3) but unfortunately the data about the statistical prevalence of this emerging pathogen were unavailable.

## 3. Discussion

In this study, the presence and occurrence of endemic and emerging TBPs in ticks collected in northeastern Italy was deeply investigated, achieving epidemiological and biological TBPs and TBDs knowledge. Our data highlight the co-circulation of nine different pathogens in the study area, with an overall TBP prevalence of 12.5% in ticks in immature stages and of 29.2% in adults. As expected, the overall TBP prevalence was higher in adults than nymphs [51]. 

At a complex level, the most prevalent pathogen reported in our study was *Rickettsia* spp. (13.7%; 5.0% in immature stages and 8.7% in adults) followed by *Borrelia* spp. (12.6%; 2.6% in nymphs and 10.0% in adults). 

Rickettsiae are known to be vertically transmitted; in fact, we found positivity in larvae with a prevalence of 1.0%. The Rickettsiae species detected in our study *(R. helvetica* and *R. monacensis)* are the main TBPs transmitted by *I. ricinus* circulating in Europe [52], and our prevalence appears to be lower than that previously reported in Italy, which ranged from 24.8% (northern Italy) to 18.4% (Central Italy) [41,53,54]. *Rickettsia helvetica* has been detected in *I. ricinus* ticks in at least 24 European countries [55], with a wide range of prevalence from 0.5% (Island of the Baltic Sea) to 66% in the Netherlands [3,56]. In northern Italy, *R. monacensis* was detected in the past with a higher prevalence (3.7–4.5%) than that reported of our study (0.3% in nymphs, −1.8% in adults) [57,58]. This could be explained when considering that *Rickettsia* spp. prevalence could be influenced by several factors, including the season, year of sampling, environment, and type of tick hosts [5]. 

The *B. burgdorferi* s.l. prevalence detected was lower compared to a previous study in northeastern Italy (4.7% in nymphs and 17.6% in adults) [38] and to other similar studies carried out in northwestern Italy (10.3–10.5%) [53,59], in the Alpine area (6.3%) in the province of Trento [60], in the Po River Valley (18.0%), and in Central Italy (20.0%) [61].

In our study, we found four genospecies (*B. burgdorferi* s.s., *B. afzelii, B. garinii*, and *B. valaisiana*). The most prevalent was *B. burgdorferi s.s*., followed by *B. afzelii*, which was different from a study conducted in northwestern Italy in which *B. garinii* and *B. afzelii* were the genospecies most frequently identified in questing ticks [53]. In Central Europe, *B. afzelii*, together with *B. garinii*, is one the most common genospecies with the highest prevalence rate [62]. Despite the first detection of *B. miyamotoi* in north Italy dating back to 2016 [38], during a retrospective study (unpublished data) *B. miyamotoi* presence was detected in Belluno province in 2011 and had been continuously present until 2017, with the exception of 2014. Considering that *B. miyamotoi* seems to be well established in the Belluno area, more attention should be paid in cases of non-specific febrile syndrome.

The most prevalent pathogen, at a species level, was *A. phagocytophilum*. The prevalence was higher in adults (7.6%) than in nymphs (2.9%) and was higher than the overall prevalence found previously in questing *I. ricinus* ticks (1.8%) in northern Italy [30]. In Europe, the infection rates range from 0.8% to 14.0% [63,64], with a greater risk of infections in eastern Europe than in western Europe [30]. The low infection rate is due to inability of *A. phagocytophilum* to be transmitted transovarially, and larvae can only be found infected after a meal on a bacteriemic host [22,65]. Accordingly, in our study no positivity for this pathogen was found in larvae.

*Neoehrlichia mikurensis* was detected in all stages with a prevalence of 0.3%, 1.6%, and 2.9% in larvae, nymphs, and adults, respectively; these results are lower compared to findings in adult ticks (10.5%) in a previous study conducted in the same area [58] but higher than data coming from northwestern Italy (2.0% in nymph and adult *I. ricinus* collected in a peri-urban park) [41]. In Europe, the prevalence of *N. mikurensis* ranged from 0.2% (Poland) [66] to 6.4% (Switzerland) [67] and in general, the median *N. mikurensis* infection rate was greater in western Europe than in Eastern Europe [8]. Despite this pathogen being discovered very recently, it is present in the Belluno area, confirming its high suitability for TBPs.

Few studies have been carried out in Italy to evaluate the distribution and prevalence of the zoonotic *Ba. venatorum*. In our study, *Ba. venatorum* was found only in nymphs with a prevalence of 0.1%, lower than the prevalence found previously in nymphs collected in northeastern Italy (2.4%) [29] and in the bordering province of Trento (3.8%) [30]. Transovarial transmission has been demonstrated [68] but in this study we did not find the pathogen in larvae, possibly because of the very low prevalence in this tick life stage. *Babesia microti* is associated with human infection [69] and it is considered a potential risk in north Italy [30], whereas it was not detected in our study.

Compared to central and eastern Europe, Italy is considered a low-risk region for TBEv. The virus seems to be restricted to areas of the northeastern part of the country [46], where it is historically endemic with a prevalence ranging from 0.2% to 2.5% [58,70,71], within the range registered in European endemic areas (0.1–5.0%) [72,73]. In our study TBE was undetected, probably because this agent has a typical distribution and presentation which is particularly scattered and circumscribed in foci; these characteristics reasonably allowed the pathogen to escape from the sampling [73]. To improve the tick positivity for TBEv detection, it would be useful to provide integrated human cases and tick monitoring surveillance [74]. 

In our study, co-infections in single adult ticks were reported in a similar percentage (1.8%) as in a previous study conducted in Italian humans (1.2%) [75]. The presence of multiple pathogens in ticks could cause co-transmission of pathogens to humans, affecting the medical diagnosis, severity of the disease, and prognosis [76,77]. In particular, in our study the dual co-infection between *Borrelia* spp. and *Rickettsia* spp. occurred, which is confirmed to be one of the most frequent co-infections as reported in Romania, Switzerland and Belgium [76,78,79]. Co-infection of *Borrelia* spp. with *Babesia* spp., suspected to enhance the severity of LB, was not observed in our study [80,81].

The majority of the ticks collected by dragging were nymphs (77.3%); this is probably a result of the dragging technique used [82,83]. Nymph and adult ticks are the most important stages in the transmission of pathogens to humans. Nymphs are the most dangerous because their small size and their activity period (spring and summer), which is the highest human outdoor activity time in temperate climates [84]. The *I. ricinus* density trend data (seasonality and distribution) reported in our study confirms what was already described in central Europe [11,85,86], although tick density in the Belluno area was lower than in other European areas [87]. The two peaks in tick abundance (May–June and October–November) correspond to the peak of TBP detection. Similarly, co-infection and multiple TBP detection in pools analyzed were recorded. Adult ticks showed a higher prevalence for each TBP compared to immature stages, where the higher TBP diversity was detected in nymphs.

All the monitored municipalities tested positive for tick presence and different species of TBPs were detected. Our results highlight the considerable variation of annual prevalence of TBPs in each municipality and detected fewer common species (e.g., *Ba. venatorum*). Regular monitoring is necessary to determine TBP prevalence of emerging or new TBPs. Tick surveillance should continue in order to detect abnormal prevalence peaks due to an imbalance between climatic conditions and reservoir availability [11,88].

The high diversity (up to nine species), proximity (four different TBPs in ticks collected within less than 100 m^2^), frequency of detection (seven different species/ sites/ years), and prevalence of TBPs characterize the Belluno province as a high-risk area for TBP transmission. Tick surveillance is necessary to assess TBP prevalence and occurrence and it allows for the identification of the different genospecies circulating in a investigated area. These data would improve prospective surveillance of TBDs in the focus area by providing more insight into the ecological and epidemiological features of TBDs [9].

Understanding and mapping *I. ricinus’* spread and prevalence is pivotal to assess the risk of TBPs spreading. Furthermore, the assessment of TBDs temporal and spatial trends would be significantly improved by social communication of messages about risk related to TBDs to policymakers, stakeholders, and the citizens [9,89]. 

## 4. Materials and Methods

### 4.1. Study Area and Sampling Method

Tick collection was conducted in 20 municipalities in the Belluno province, Veneto Region (northeastern Italy). Sampling activity was performed in sites with human frequency for both work and leisure activities, such as parks, hiking trails, start of climbing routes, and peri-urban recreational areas (Table A1).

In order to cover the two peaks in tick activity (spring and autumn), sampling activity was carried out each year from 2011 to 2017 and from April to November [46,86].

The technique used by forest rangers and the local health unit for the collection of questing ticks was ‘tick dragging’, using a 1 m^2^ white flannel cloth dragged across the top of the vegetation or forest floor in a designated transept (100 m) and regularly checking for the presence of ticks [58].

Collected ticks were identified based on morphological keys [90,91], and were pooled and stored at –80 °C until molecular analysis for the pathogen identification.

### 4.2. Molecular Analysis

Single adult ticks, pooled nymphs (maximum 13 specimens per pool), and larvae (maximum 22 specimens per pool) were homogenized in 600 μL of Phosphate Saline Buffer (PBS) with a 5 mm bead (Qiagen) using the instrument TissueLyser II (Qiagen). Then, 150 μL of supernatant was used for the nucleotide extraction using the All Prep DNA/RNA Mini Kit (Qiagen, Valencia, CA, USA), according to the manufacturer’s instructions, and then kept frozen at −80 °C. RNA was screened for the detection of TBE virus using a TaqMan Real-Time PCR, as described elsewhere [92]. DNA was screened for the detection of *B. burgdorferi s.l.* and *N. mikurensis* using traditional PCR, as described elsewhere [93,94], and by using in-house SYBR Green Real-Time PCR (rtPCR) for the detection of *A. phagocytophilum*, *Rickettsia* spp., and *Babesia* spp. The target genes, primers used, and related references are listed in Table A2. Each reaction was carried out in a total volume of 20 μL, containing 10 μL of Quanti-Fast SYBR Green PCR Master mix 2× (Qiagen GmbH, Hilden, Germany), sense and reverse primers (concentration reported in Table 1), and 3 μL of DNA. Amplifications were performed in a StepOnePlus™ instrument (Applied Biosystems, Foster City, CA, USA). The thermal profile consisted of 5 min of activation at 95 °C, followed by 40 cycles at 95 °C for 15 s (denaturation), specific annealing temperature (Table 1) for 30 s (annealing), and 60 °C for 30 s (extension). Following amplification, a melting curve analysis was performed by slowly raising the temperature of the thermal chamber from 60 °C to 95 °C to distinguish between specific and non-specific amplification products. To ensure the effectiveness of DNA and RNA extraction, a PCR targeting the 18S rRNA gene internal control and a TaqMan Real-Time PCR targeting the 16S rRNA gene were applied, respectively [92,95]. Negative (sterile water) and positive controls (DNA or RNA of pathogens) were included in each run. PCR products were examined on 7% acrilamide gels stained with SYBR Gold® Nucleic Acid Gel Stain 1× (Thermo Fisher Scientific) and visualized on a Molecular Imager® Gel DocTM XR System (Biorad). DNA amplification products were directly sequenced for species identification. The amplicons were purified and sequenced in both directions with the same primers used for PCR and qPCR, using a 16-capillary ABI PRISM 3130xl Genetic Analyzer (Applied Biosystems, Foster City, CA, USA). Sequences were aligned and compared with those available in GenBank by Basic Local Alignment Search Tool (BLAST—http://blast.ncbi.nlm.nih.gov/Blast.cgi; accessed on 5 January 2018).

**Figure A1:**
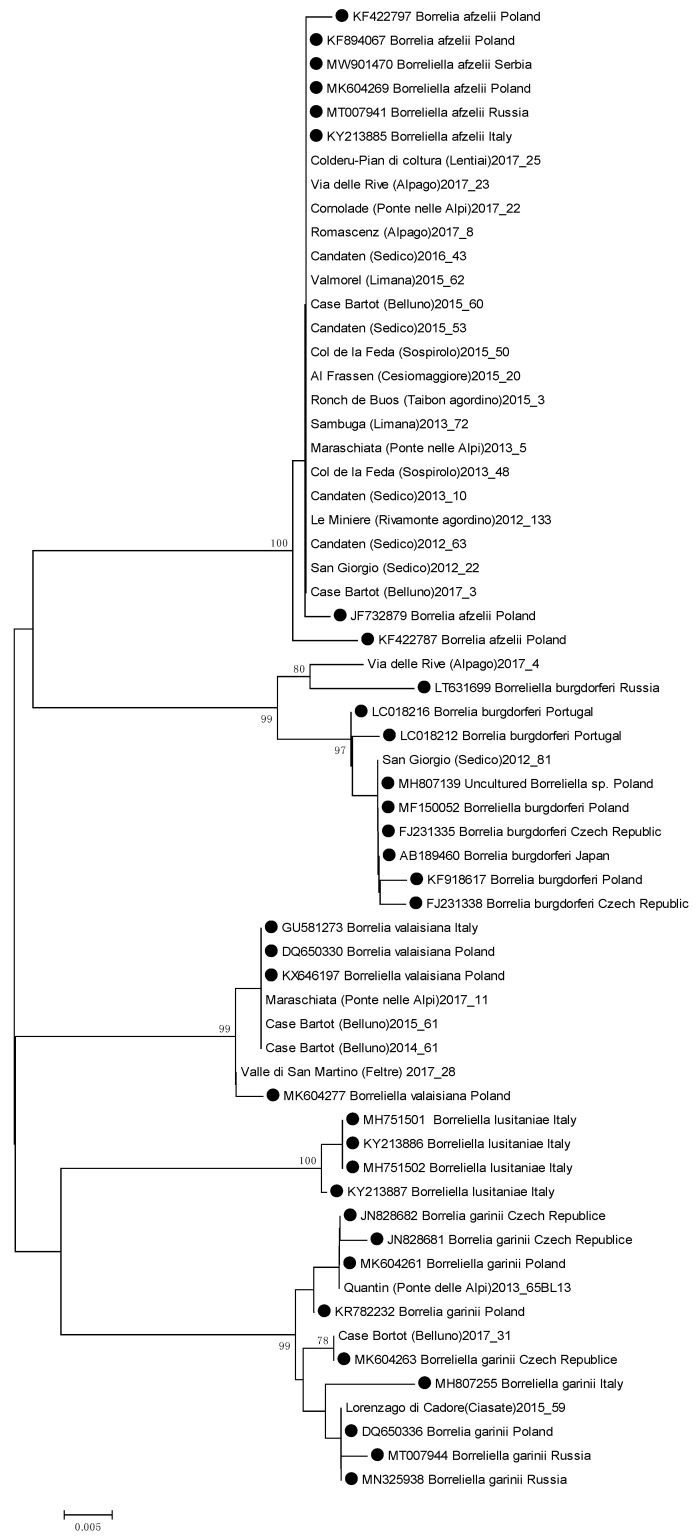
Phylogenetic tree based on *Fla* gene partial sequences of *Borrelia burgdorferi* sensu lato. Sequence dataset was analyzed using MEGA v6.0 [103]; the tree was constructed using the neighbor-joining (NJ) method and bootstrap analysis (1000 replicates) based on the ClustalW algorithm. Significant bootstrapping values (>70%) are shown on the nodes. The tree shows the representative sequences of this study based on years and sites. ● Reference sequences from Genbank.

**Figure A2:**
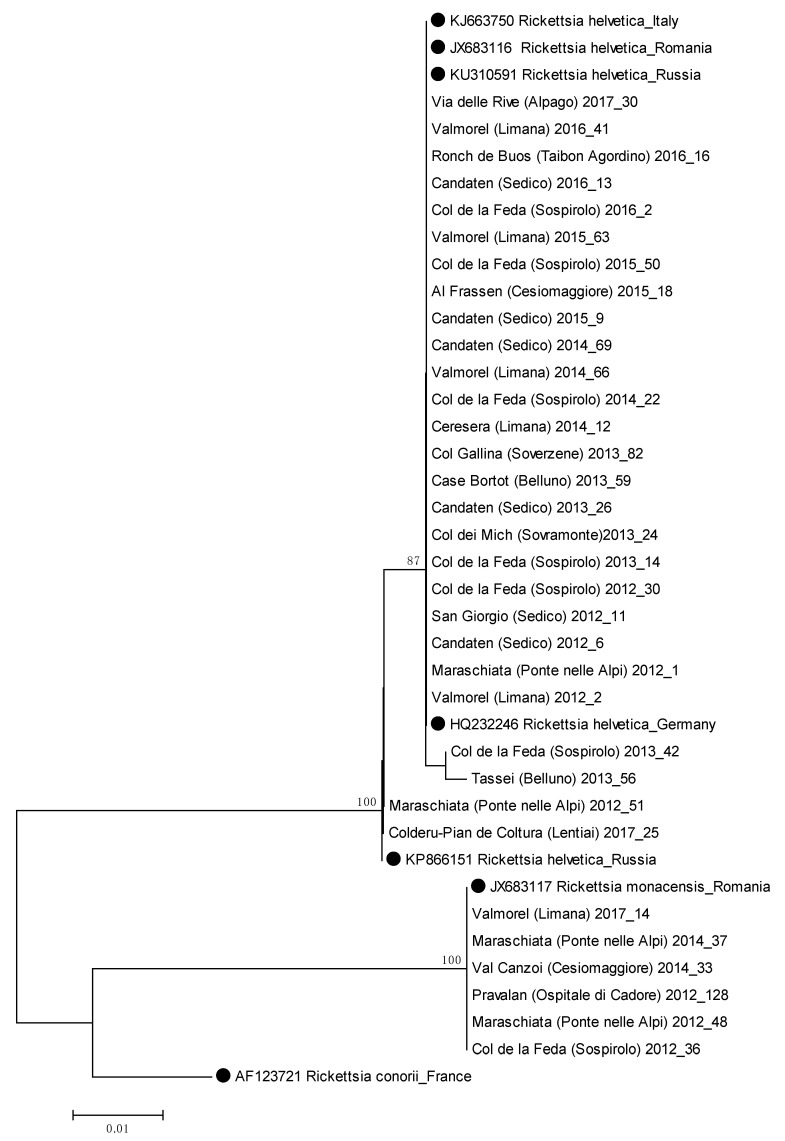
Phylogenetic tree based on *rOmpB* gene partial sequences of *Rickettsia* spp. Sequence dataset was analyzed using MEGA v6.0 [103]; the tree was constructed using the neighbor-joining (NJ) method and bootstrap analysis (1000 replicates) based on the ClustalW algorithm. Significant bootstrapping values (>70%) are shown on the nodes. *Rickettsia conorii* was used as outgroup. The tree shows the representative sequences of this study based on years and sites. ● Reference sequences from Genbank.

Phylogenetic analysis related to *Borrelia burgdorferi* genospecies and the Rickettsia species identified in this study were performed using the representative sequences, from 2012 to 2017, based on years and site. Phylogenetic trees (Figure A1 and Figure A2) can be inserted into Appendix A.

The DNA of a pool subsample (343/596 pools, 57.5%) was screened for the presence of *Borrelia miyamotoi* using a Traditional PCR targeting ~900 bp of the glpQ gene [17].

### 4.3. Statistical Analysis

Tick density was calculated as average number of ticks per 100 m^2^ [96]. Pool positivity rate (PPR), corresponding to the number of positive pools/total pool of ticks examined, was calculated (pool positive for more than one TBPs count as 1). Estimate prevalence (hereafter named Prevalence) for variable pool size together with uncertainly intervals (hereafter named confidence interval, CI) for each TBP and for TBP in general in each municipality (both annually and mean) were calculated through PoolTestR package [97]. Estimation of prevalence based on presence/absence tests on pooled samples was obtained using the script codes provided by PoolTestR package (see the package description for details). All data cleaning and preparation and graphics were conducted using R statistical software version 4.1.0 [98] and the package Tidyverse [99]. Maps and spatial data manipulation were carried out using ESRI ArcMap (ArcGIS Desktop: Release 10.5.1. Redlands, CA, USA: Environmental Systems Research Institute. Copyright© 1999–2017).

## 5. Conclusions

Continuous tick surveillance and TBP screening is of pivotal importance and should be maintained to collect updated data on TBP prevalence, occurrence of different genospecies circulating in the province, and to estimate the risk related to TBPs in the population. These aspects have been highlighted through this study, confirming that Belluno province is highly endemic to TBPs, with co-circulation of up to nine different TBPs. Furthermore, these data should be used to carry out risk communication campaigns aimed at sharing knowledge about different tick species present in the area, as well as the pathogens they can transmit and preventive measures against infectious tick bites. Furthermore, these data should be used to carry out risk communication campaigns aimed at implementing preventive measures against infectious tick bites. In conclusion, a multidisciplinary ‘One Health’ approach is crucial to perform eco-epidemiological research and surveillance specifically focused on the occurrence of ticks, their infection with pathogenic microorganisms, as well as on the presence of tick maintenance and reservoir vertebrate hosts.

## Figures and Tables

**Table 1:** Number (N) of adults, nymphs, and larvae of *I. ricinus* collected monthly during the seven years of surveillance (2011–2017) in Belluno Province and mean number of ticks collected per sampling.

Month	Years	N Sampling	N Adults	N Nymphs	N Larvae	N Total Ticks	N Mean/Sampling
April	2012, 2014	16	26	152	3	181	11.3
May	2011–2015	40	79	722	0	801	20.0
June	2011–2017	38	124	533	0	657	17.3
July	2011–2017	19	14	105	50	169	8.9
August	2013, 2016–2017	6	2	13	1	16	2.7
September	2011–2017	33	11	202	137	350	10.6
October	2011, 2013–2017	29	15	258	132	405	14.0
November	2011, 2013, 2015	5	4	77	8	89	17.8
Total	2011–2017	186	275	2062	331	2668	14.3

**Table 2:** Monthly number of total and TBP-positive pools and pool positivity rate (PPR) in Belluno province (2011–2017).

Month	Years	N Positive Pool/N Total Pool	PPR (%)
April	2012, 2014	14/48	29.2
May	2011–2015	76/174	43.7
June	2011–2017	73/192	38.0
July	2011–2017	8/39	20.5
August	2013, 2016–2017	2/8	25.0
September	2011–2017	23/58	39.7
October	2011, 2013–2017	27/61	44.3
November	2011, 2013, 2015	10/16	62.5
Total	2011–2017	233/596	39.1

**Table 3:** Number of positive pools per each TBP, prevalence, and associated 95% confidence interval (CI 95%) in adults, pooled larvae, and nymphs of ticks collected in Belluno province from 2011 to 2017.

Stage	TPBs	Number of Positive Pool	Prevalence	CI 95% (CI Low-CI High)
**Larvae**	*Rickettsia helvetica*	3	1.0	0.2–2.5
	*Neoehrlichia mikurensis*	1	0.3	0.0–1.3
	**Total**	**4**	**1.3**	**0.1–1.9**
**Nymphs**	*Rickettsia helvetica*	67	3.7	2.9–4.7
	*Rickettsia monacensis*	7	0.3	0.1–0.7
	*Borrelia afzelii*	33	1.7	1.2–2.3
	*Borrelia burgdoferi* s.s.	10	0.5	0.2–0.9
	*Borrelia garinii*	5	0.2	0.0–0.5
	*Borrelia valaisiana*	4	0.2	0.0–0.5
	*Anaplasma phagocytophilum*	54	2.9	2.2–3.8
	*Neoehrlichia mikurensis*	31	1.6	1.1–2.2
	*Babesia venatorum*	1	0.1	0.0–0.3
	**Total**	**212**	**11.2**	**0.9–1.8**
**Adults**	*Rickettsia helvetica*	19	6.9	4.2–10.6
	*Rickettsia monacensis*	5	1.8	0.6–4.2
	*Borrelia afzelii*	7	2.6	1.0–5.2
	*Borrelia burgdoferi s.s.*	18	6.6	3.9–10.2
	*Borrelia valaisiana*	2	0.8	0.1–2.6
	*Anaplasma phagocytophilum*	21	7.6	4.8–11.4
	*Neoehrlichia mikurensis*	8	2.9	1.3–5.7
	**Total**	**80**	**29.2**	**2.3–7.1**

Note: *Babesia venatorum* screening started in 2013.

**Table 4:** Co-infection detected in *I. ricinus* in single adults.

TBPs	Sex
*Rickettsia helvetica + Borrelia afzelii*	M
*Rickettsia helvetica + Anaplasma phagocytophilum*	F
*Rickettsia monacensis + Anaplasma phagocytophilum*	M
*Rickettsia monacensis + Neoehrlichia mikurensis*	M
*Borrelia burgdoferi s.s. + Anaplasma phagocytophilum*	F

Note: F = female and M = male.

**Table 5:** Multiple detection of TBPs in pooled *I. ricinus* nymphs (combination of three and two TBPs in the first and second box, respectively).

TBPs Combination	N Pool
*Rickettsia helvetica + Borrelia garinii + Anaplasma phagocytophilum*	1
*Rickettsia helvetica + Borrelia burgdoferi s.s. + Anaplasma phagocytophilum*	1
*Rickettsia helvetica + Borrelia afzelii + Neoehrlichia mikurensis*	2
*Rickettsia helvetica + Borrelia afzelii + Anaplasma phagocytophilum*	2
*Rickettsia helvetica + Anaplasma phagocytophilum + Neoehrlichia mikurensis*	1
*Borrelia afzelii + Anaplasma phagocytophilum + Neoehrlichia mikurensis*	1
*Rickettsia helvetica + Rickettsia monacensis*	1
*Rickettsia helvetica + Borrelia afzelii*	9
*Rickettsia helvetica+ Borrelia burgdoferi s.s.*	1
*Rickettsia helvetica + Borrelia valaisiana*	1
*Rickettsia helvetica + Anaplasma phagocytophilum*	12
*Rickettsia helvetica + Neoehrlichia mikurensis*	5
*Rickettsia monacensis + Borrelia afzelii*	1
*Rickettsia monacensis + Neoehrlichia mikurensis*	1
*Borrelia afzelii + Anaplasma phagocytophilum*	2
*Borrelia afzelii + Neoehrlichia mikurensis*	4
*Borrelia burgdoferi s.s. + Anaplasma phagocytophilum*	4
*Borrelia burgdoferi s.s. + Neoehrlichia mikurensis*	1

**Table 6:** Annual prevalence, mean prevalence, 95% confidence interval, and number of TBPs positive/total pools in each monitored municipality in Belluno province (2011–2017).

Municipality	Annual TBPs Prevalence							Mean TBPs Prevalence	CI 95% (CI Low-CI High)	Number of Positive/Total Pools
	**2011**	**2012**	**2013**	**2014**	**2015**	**2016**	**2017**			
Alpago	−	−	−	−	−	−	44.4	44.4	16.5–75.1	4/8
Belluno	−	0.0	28.8	5.3	19.4	18.4	23.7	13.1	6.4–22.8	9/34
Cencenighe	−	−	0.0	−	−	−	−	0.0	0.0–85.3	0/1
Cesiomaggiore	−	0.0	−	13.9	13.9	0.0	−	10.8	5.0–19.7	8/21
Feltre	−	−	−	−	−	−	15.5	15.5	1.0–28.9	1/4
La Valle Agordina	−	100.0	−	−	−	−	−	100.0	2.6–100.0	1/1
Lentiai	30.1	−	−	−	−	−	1	38.1	6.4–93.5	2/3
Limana	−	100.0	9.1	11.0	7.6	6.7	7.3	8.6	4.8–13.9	13/35
Longarone	−	−	−	0.0	−	−	–	0.0	0.0–61.7	0/2
Lorenzago di Cadore	−	−	−	−	19.3	33.3	0.0	19.7	3.4–52.4	2/7
Ospitale di Cadore	−	50.0	−	−	−	–	−	50.0	3.8–96.2	1/2
Ponte nelle Alpi	−	27.8	7.1	20.3	19.3	0.0	25.0	14.5	7.5–24.3	10/36
Quero-Vas	−	−	−	15.4	−	−	−	15.4	2.7–40.4	2/12
Rivamonte Agordino	−	12.6	−	−	−	−	−	12.6	0.8–45.6	1/4
Sedico	17.6	15.7	19.9	33.0	21.2	13.6	−	17.8	14.4–21.6	90/174
Sospirolo	9.2	5.1	6.7	50.4	7.7	9.8	−	8.3	6.7–10.2	85/239
Soverzene	−	−	1.0	0.0	−	−	-	33.3	2.3–83.9	1/2
Sovramonte	−	−	24.0	−	0.0	−	−	9.6	0.6–36.7	1/4
Taibon Agordino	−	−	−	−	15.9	33.3	−	21.7	3.9–54.6	2/4
Tambre	−	−	0.0	−	0.0	−	−	0.0	0–38.1	0/3
**Total**	**19.0**	**31.1**	**24.5**	**16.6**	**12.4**	**14.4**	**30.8**	**21.7**	**4.4–50.2**	**233/596**

Note: −= not monitored; CI = confidence interval.

## Data Availability

All data are reported in the manuscript or in the Appendix A.

## Appendix A

**Table A1:** Geographical details of monitored sites in Belluno province during 2011–2017.

Municipality	ID Site	Altitude	Coordinate N	Coordinate E
Alpago	1	420	46.136444	12.367389
Alpago	2	410	46.139861	12.351278
Belluno	3	NA	NA	NA
Belluno	4	700	46.182500	12.195000
Belluno	5	500	46.174394	12.240835
Belluno	6	377	46.153918	12.266074
Belluno	7	590	46.068515	12.245752
Cencenighe	8	785	46.341956	11.971707
Cesiomaggiore	9	800	46.144200	11.952100
Cesiomaggiore	10	765	46.128226	11.949862
Feltre	11	550	46.060320	11.913008
La Valle Agordina	12	792	46.274276	12.058691
Lentiai	13	740	46.032543	12.009134
Limana	14	532	46.092943	12.221204
Limana	15	795	46.057335	12.240001
Limana	16	800	46.053956	12.228388
Longarone	17	470	46.273525	12.310835
Lorenzago di Cadore	18	750	46.478346	12.448012
Ospitale di Cadore	19	550	46.319769	12.321676
Ponte Nelle Alpi	20	762	46.123483	12.298094
Ponte nelle Alpi	21	540	46.130056	12.310583
Ponte Nelle Alpi	22	550	46.156157	12.287511
Ponte Nelle Alpi	23	710	46.143404	12.284754
Ponte nelle Alpi	24	1000	46.106111	12.287778
Quero-Vas	25	500	45.944154	11.885902
Rivamonte Agordino	26	973	46.257617	12.040636
Rivamonte Agordino	27	681	46.268299	12.031582
Sedico	28	420	46.199303	12.125336
Sedico	29	340	46.124025	12.131986
Sedico	30	360	46.129335	12.106427
Sospirolo	31	580	46.158888	12.057320
Sospirolo	32	1681	46.167946	12.013621
Soverzene	33	396	46.206493	12.302831
Soverzene	34	720	46.214236	12.311723
Sovramonte	35	790	46.066889	11.782164
Sovramonte	36	1200	46.067969	11.830914
Taibon Agordino	37	700	46.308424	12.010278
Tambre	38	1050	46.104861	12.441278
Tambre	39	806	46.121906	12.390455

Note: NA = not available.

**Table A2:** Target genes, primer concentration, annealing temperatures, and related references of primers used for the detection of *A. phagocytophilum*, *Rickettsia* spp., and *Babesia* spp.

Pathogens	Target Gene	Primer	Sequence	Ta (°C)	PC (µM)	Fragment Lenght (bp)	*Reference*
***A. phagocytophilum***	msp2	msp2-3f msp2-3r	5′-CCAGCGTTTAGCAAGATAAGAG-3′	56 °C	0.05	334	[100]
			5′-GMCCAGTAACAACATCATAAGC-3′				
***Rickettsia*** **spp.**	ompB	rompB OFm rompB ORm	5′-GTA ACC GGA ART AAT CGT TTC GT-3′	58 °C	0.1	511	modified after [101]
			5′-GCT TTA TAA CCA GCT AAA CCR CC-3′				
***Babesia*** **spp.**	18S rRNA	BJ1	5′-GTC TTG TAA TTG GAA TGA TGG-3′	60 °C	0.1	500	[102]
		BN2	5′-TAG TTT ATG GTT AGG ACT ACG-3′				

Note: Ta = annealing temperature; PC = primer concentration: msp2 = major surface protein 2; ompB = outer membrane protein B; 18S rRNA = 18S ribosomal RNA.

**Table A3:** *Borrelia miyamotoi* pool positivity results during a retrospective study in a subsample of ticks collected in Belluno province (2011–2017).

N Pool	Year	Municipality	ID Site	Tick Stadium and Sex	Specimen Number
1	2011	Sospirolo	31	Nymphs	10
2	2011	Sospirolo	31	Adult Male	1
3	2011	Sospirolo	31	Adult Female	1
4	2011	Sedico	29	Nymphs	6
5	2012	Sedico	28	Nymphs	10
6	2012	Ospitale di Cadore	19	Adult Female	1
7	2013	Sospirolo	31	Nymphs	10
8	2013	Sospirolo	31	Nymphs	10
9	2015	Limana	16	Nymphs	10
10	2016	Sospirolo	31	Nymphs	11
11	2016	Sedico	28	Nymphs	10
12	2016	Sedico	28	Nymphs	10
13	2017	Limana	16	Nymphs	11
14	2017	Alpago	1	Adult Male	1
15	2017	Limana	16	Nymphs	7
